# Post-Pandemic Stress Disorder as an Effect of the Epidemiological Situation Related to the COVID-19 Pandemic

**DOI:** 10.3390/healthcare10060975

**Published:** 2022-05-24

**Authors:** Daria Łaskawiec, Mateusz Grajek, Patryk Szlacheta, Ilona Korzonek-Szlacheta

**Affiliations:** 1 Department of Prevention of Metabolic Diseases, Faculty of Health Sciences in Bytom, Medical University of Silesia, 41902 Katowice, Poland; d201081@365.sum.edu.pl (D.Ł.); ikorzonek@sum.edu.pl (I.K.-S.); 2 Department of Public Health, Faculty of Health Sciences in Bytom, Medical University of Silesia, 41902 Katowice, Poland; 3 Department of Toxicology and Health Protection, Faculty of Health Sciences in Bytom, Medical University of Silesia, 41902 Katowice, Poland; pszlacheta@sum.edu.pl

**Keywords:** post-pandemic stress disorder (PPSD), post-traumatic stress disorder (PTSD), SARS-CoV-2, COVID-19, pandemic stress and anxiety

## Abstract

According to many experts in the fields of psychology and psychiatry, the destabilization resulting from the coronavirus pandemic may not be as noticeable now as it will be after the pandemic period is over. Undoubtedly, the fact that the surrounding reality is standardized and normalized by many at present contributes to this. In the opinion of many researchers, the scale and degree of trauma experienced by society will only be noticed by many once the pandemic is over. Many also suggest that we will experience post-pandemic stress disorder. This literature review aims to bring together in one place the information that speaks to the nature of the problem, which is post-pandemic stress disorder. The main sections of the paper deal with exposure to the disorder in the general population and a review of the current literature on the subject. The second section deals with a group of medical personnel who are on the direct frontline in the fight against the COVID-19 pandemic; it is assumed here that they are those who are at much higher risk of developing post-pandemic stress disorder.

## 1. Introduction

Acute coronavirus disease (COVID-19) has been considered a worldwide pandemic as a result of rapid viral transmission. Although the disease is mild in most people, some patients (especially the elderly and/or those with chronic comorbidities) may develop severe bilateral pneumonia, acute respiratory distress disorder, and subsequent multiorgan dysfunction, which may lead to death [1,2,3]. Therefore, the fear of infection and especially the severity of the disease itself and death has undoubtedly become the cause of generalized anxiety and fear in many people. This anxiety may be dictated not only by fears for one’s health, but also for the health and lives of loved ones. The COVID-19 pandemic has undoubtedly affected the mental health of the population to a great extent and even left a permanent mark on it [4,5]. Because the fear of something unknown, which is certainly the pandemic of a new coronavirus announced by the World Health Organization (WHO) on 11 March 2020, contributes to the manifestation of anxiety symptoms in society, it thus initially worsens the state of mental health, even of healthy people [6,7].

The World Health Organization updates the statistics related to the coronavirus daily. Based on them, from 3 January 2020 to 17 April 2022, more than 6.2 million people died worldwide due to COVID-19 [8]. In light of the given statistics and information, WHO pays great attention to the possibility of the spread of the disease and the transmission of the infection from patients to healthcare workers. Referring to medics, it is not uncommon for them to be under great psychological pressure associated with being on the front line in the fight against the coronavirus. The existing stressors at work, such as exposure to the disease, the possibility of transmitting the infection to their loved ones, dealing with the death of hospitalized patients, the pressure of prolonged on-call duty, and spending many hours at work beyond their capabilities, as well as the lack of supplies of personal protective equipment, have significantly contributed to the development of stress and anxiety [9,10]. For this reason, a much more frequent and increasingly characteristic phenomenon occurring in a much higher percentage of medics than before the pandemic is professional burnout disorder, commonly referred to in the era of the pandemic as “covidian burnout” (from COVID-19 burnout) [11,12]. It is a psychological disorder resulting from the continuous exposure to emotional and/or psychological stressors in the work environment. The disorder consists of three elements, which are its essence. The first is emotional exhaustion, associated with an extreme lack of energy to live. The second is depersonalization, involving a sense of impersonality and the development of negative feelings. Additionally, the third is a reduction in the evaluation of personal achievements, associated with a subjective lack of professional effectiveness. Undoubtedly, this disorder harms the healthcare system, because the work efficiency of medics affected by this disorder drastically decreases. The absenteeism phenomenon then increases, as well as the likelihood of medical staff turnover, which undoubtedly translates into the quality of health care for sick patients [13,14].

Due to the possible severe course of the disease, the WHO, as soon as the first cases of coronavirus appeared, announced official recommendations for people to minimize the risk of infection. For this reason, the first restrictions and limitations were introduced in Poland in March 2020, as a result of which the whole society was deprived of access to various services [15,16]. The daily lives of many people have significantly changed and the ongoing pandemic has become a challenge not only for the health sector, but also for the whole economy, finance, education, tourism, culture, and public health in general [16,17]. It has led to negative changes in many areas of life, the consequences of which society continues to bear today. In turn, the collective effect of these factors has shown a destructive impact on the mental health of society worldwide.

Because of the above, this literature review aims to bring together in one place the information that speaks to the essence of the problem, which is post-pandemic stress disorder. The main sections of the paper deal with the exposure to the disorder in the general population and a review of the current literature in this area. The second section deals with a group of medical personnel as those who are on the direct frontline in the fight against the COVID-19 pandemic; it is assumed here that they are much more vulnerable to the occurrence of post-pandemic stress disorder.

## 2. Review Methodology

### 2.1. Review Procedure

The review was conducted according to good practices associated with conducting similar reviews. The literature items were searched by a team of researchers (authors) along with a library staff member trained in literature searching and EBM (evidence-based medicine) and HTA (health technology assessment). An initial search for items consistent with the topic and purpose of the review was conducted to determine the field of study. After reviewing the existing data, a keyword package was selected that seemed the most relevant and consistent with the review topic.

### 2.2. Eligibility Criteria

The primary eligibility criteria were the language of publication, years of research or review, publication status, and whether the authors were specialists in their field (or had other publications in a similar field), and lack of external finance. Regarding the language, English-language articles were selected because this language seems to be universal in the scientific community. In addition, articles published after 2019 were included to ensure that the topic covered was strictly related to the COVID-19 epidemiological situation. Furthermore, articles that were available in full text on an open-access basis were selected.

### 2.3. Search Strategy

**Figure 1:**
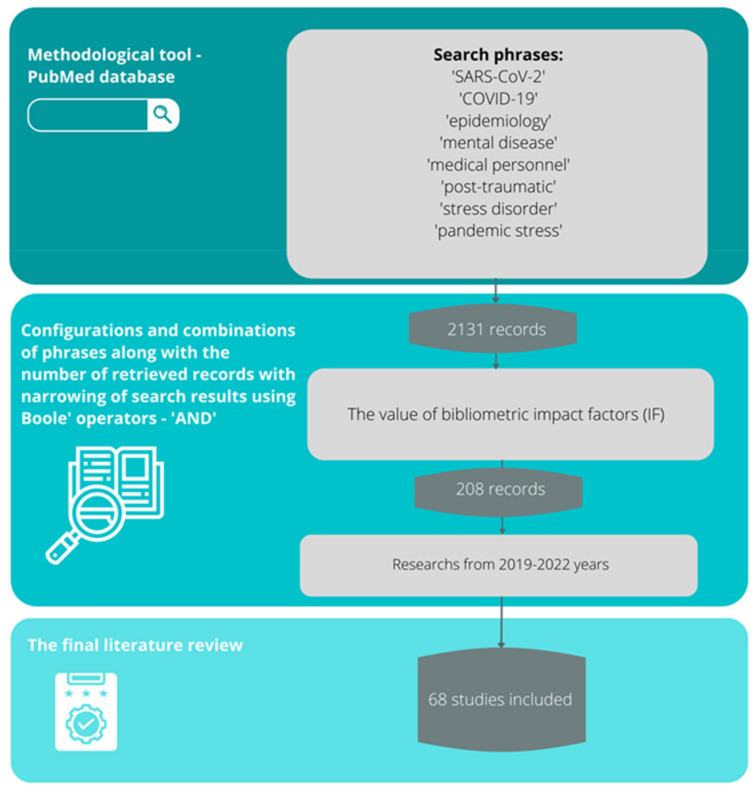
Review methodology—handling of the literature selection.

A review of the scientific evidence was conducted based on the available literature by entering sample phrases (consistent with the MeSh dictionary) with Boole operators, logical operators (and, or, not), and special characters: SARS-CoV-2, COVID-19, epidemiology, mental illness, medical personnel, post-traumatic stress disorder, pandemic stress (and various combinations thereof) using the methodological tool of the PubMed database. The PubMed database in this regard seemed most appropriate due to the fact that it is a methodological tool that allows searching for articles available in multiple scientific databases (such as Medline or Embase). Its use provides the opportunity to meet all expectations from the review (transparency, clarity, comprehensiveness, focus, uniformity, accessibility, coverage of the entire topic). Only English-language articles published between 2019 and 2022 were included in the search. The literature review primarily focused on determining the prevalence in the general population and medical personnel of mental-health deterioration due to the COVID-19 pandemic through the occurrence of symptoms, such as, for example, anxiety, depression, extreme fatigue, or the emergence of occupational burnout. In addition, the occurrence of more distant consequences, such as PTSD, was also assessed, which may be due to the chronicity of the period of the COVID-19 pandemic, as well as its long-term impact on the psyche of medical professionals (Figure 1).

### 2.4. Sources Selection

Originally, 2131 papers matched according to the above criteria were analyzed, but during the analyses, 208 papers were found to be directly relevant to the topic of the review, of which 68 were selected that were the highest indexed in terms of the bibliographic index. The accuracy, objectivity, validity, and relevance of the evidence was tested using questions consistent with the GRADE scale: Is the information reliable? Is the information free of mistakes? Has the information been properly substantiated? Is it possible to verify the information against other reliable sources? Who are the authors? Are they qualified to present information on the topic? Are they affiliated with reputable institutions working on the issue? Is the data source peer-reviewed? For what purpose was the information presented? Is the information an evidenced-based fact or constitutes an opinion? Is the information subject to risk? Can this risk be estimated? When was the information published? Is the information current or outdated? Is the timeliness relevant to the issue at hand? Does the information cover the entire issue? Does the information contain background data or does it explore the issue in depth?

### 2.5. Critical Appraisal

In critically evaluating the sources, attention was paid to whether the articles appeared in peer-reviewed journals (by at least two reviewers) and whether they had an impact factor. As described above, 68 sources were eligible for final review. A limitation of the method adopted was primarily the exclusion of sources written in a language other than English. In addition, IF has many well-documented drawbacks as a research assessment tool, and therefore is not the best way to evaluate the quality of individual research articles. Nevertheless, it was chosen because it is a synthetic indicator of a source’s impact on the field of science, and a journal that has it can more likely claim to be publishing credible scientific evidence. The review did not include so-called “grey literature”, i.e., literature that has not been subjected to the review process or that is internal to the university (for example, theses, conference reports, government leaflets, newsletters). Despite their multiple values, these sources are characterized by a high risk of containing outdated knowledge.

## 3. Post-Traumatic Stress Disorder versus Post-Pandemic Stress Disorder

Tedros Adhanom, the Director of the WHO, in one of the conferences defined that the whole world has to prepare for the so-called “mass trauma” caused by the current epidemiological situation. For this reason, the development of support for the mental health and psychosocial well-being of all people reveals a very important role in the development of recovery plans [18]. However, it is worth noting that these negative effects may be felt by individuals even after the end of the pandemic, as they may lead them to develop depression or the so-called post-pandemic stress disorder (PPSD) [19].

The term post-pandemic stress disorder was introduced by psychotherapist Owen O’Kane in 2021. Although it is not an independent disease entity as of today, it is a serious problem that many people face, often without even being aware of it. The term has not been introduced to the international classification of mental and behavioral disorders ICD-10, however, this may shortly occur. According to its creator, the term post-traumatic stress disorder refers to the so-called Post-traumatic stress disorder (PTSD) [19,20]. Post-traumatic stress disorder is a mental disorder that occurs as a result of exposure to a traumatic, extreme, and stressful event. The event exceeds the individual’s ability to adapt and cope with stress. In the case of PTSD, this is most often a single large harrowing event, such as seeing people dying in a war, experiencing rape or sexual abuse, being diagnosed with a fatal disease, or other types of major disasters [21]. In contrast, in the case of PPSD, the resulting trauma is due to experiencing and being affected by several smaller distressing experiences. These may include a fear of infection, exposure to quarantine and isolation, fear of job loss, lockdown, loneliness, and loss of social life [19,20]. 

In many countries around the world, there are currently two classifications of post-traumatic stress disorder on which the diagnosis of PPSD is based. These are DSM-5 (2013) and ICD-10. Based on the Diagnostic and Statistical Manual of Mental Disorders DSM-5 of the American Psychiatric Association (APA), the DSM-5 classification was developed with several criteria [21,22]. Criterion A, for example, is related to a stressor and requires “exposure to actual or threatened death, serious injury, or sexual violence” through direct exposure, being a witness, or reporting that one’s relative/close friend has been exposed to trauma. A distinction is also made between so-called indirect exposure occurring, for example, in medical professionals during their professional work. Criterion B is the presence of at least one of the listed symptoms of “psychological intrusion” in the affected person. Examples are recurrent and unwanted memories, flashbacks, and high emotional stress. Criterion C is the avoidance of stimuli related to the traumatic event by avoiding internal (personal feelings, sensations) as well as external (places, people, objects) reminders [23,24]. Criterion D is primarily negative changes in a person’s mood and cognition that are correlative with the traumatic event. Criterion E includes changes in reactivity and arousal. Criterion F is the duration of symptoms. For a diagnosis of PTSD, symptoms must last longer than 1 month. Criterion G is related to the functional significance of PTSD, such as leading to chronic suffering and/or disability in many aspects of life. Criterion H is the so-called exclusion of the effects of other substances, especially psychoactive substances, and the absence of the presence of other medical entities that may erroneously indicate the development of PTSD [25]. 

The diagnostic criteria according to the ICD-10 classification are similar to the DSM-five classification described, although some differences between them are noticeable. The ICD classification tends to show a relatively less complex and simpler position for psychiatric diagnoses compared to the DSM classification discussed earlier [26]. Nevertheless, the core of the ICD classification remains the reaction to a highly stressful event and the recurrence of the stressor in persistent so-called “flashbacks”. All guidelines are based on symptoms of anxiety and fear, which include recurrent re-experiencing of a specific traumatic event. The avoidance of reminders and the prospect of danger are also important. Indeed, a key component of PTSD is re-experiencing memories of the harrowing event in the present [27]. For example, the ICD-10 classification lists “severe stress reaction and adjustment disorder” in category F43. This is the only diagnostic category that is formulated as having to do with psychotraumatic impact. In addition, the ICD-10 classification also distinguishes between “acute stress reaction” and “post-traumatic stress disorder” [28].

Although COVID-19 does not tentatively fit into models of post-traumatic stress disorder, both according to the DSM-5 and ICD-10 classifications and their respective diagnostic criteria, published studies increasingly describe traumatic stress symptoms arising from an ongoing stressor, such as a coronavirus pandemic. Current models focus very much on the direct exposure of trauma to certain types of life-threatening events. However, traumatic stress reactions to future indirect exposure to trauma and non-criterion A events exist, which suggests that COVID-19 is also a traumatic stressor that may lead to PTSD symptomology [28,29].

The symptoms of PPSD and PTSD are very similar but may manifest differently in each person. Nevertheless, the most typical symptoms include: feeling of fear and anxiety, recurrent and intrusive thoughts, presence of negative emotions, social withdrawal, sleep disorders, change in eating behavior, feeling of powerlessness, and dissociative disorders [20,28]. Symptoms identical to a diagnosis of PTSD also include re-experiencing the traumatic event and avoiding any experiences that trigger memories of the event. People experiencing PTSD often avoid even the smallest stimuli referring to the traumatic event. These symptoms often contribute to discomfort in public encounters and a growing distrust of interpersonal relationships and generalized fear. Some individuals may also experience identity diffusion, consisting of a distorted self-image [30]. All of the symptoms discussed can last from at least a few weeks to even a few years in a small percentage of cases and can have a serious impact on an individual’s life [28]. 

Post-pandemic stress disorder is currently being promoted by the author of the term, psychotherapist O’Kane, who is working towards the recognition of a new mental health condition, PPSD. He strongly emphasizes that in the case of PPSD, it is more important to find the root cause of the symptoms than to try to cope with the symptoms themselves. He says that if the underlying fear is trauma caused by a stressor from the COVID-19 pandemic, then it is the trauma itself that needs to be tackled first, otherwise, the symptoms will recur. Because the trauma is not resolved and in some way closed by people experiencing PPSD, then their organism will constantly be in the so-called danger mode, as a result of which the severe and intrusive symptoms will be constantly felt [19,20].

To summarize the theme of PTSD and PPSD, the global pandemic crisis has triggered a range of different emotional and psychological responses, both collective and individual, which we can observe daily among people living alongside us. However, the pandemic may prove to be a particularly serious stressor for patients who have survived COVID-19 and healthcare workers. Moreover, the traumatic events related to the pandemic that the public has continuously experienced over the past years have had a lasting impact on their psyche, and therefore the need to care for their mental health should be considered to prevent PPSD [30,31].

## 4. The Psychosocial Context of the COVID-19 Pandemic—Review of Conducted Research

Relating the above data to the general population, several studies on the impact of the COVID-19 epidemiological situation on mental health can be found in the global literature. One national study of the UK population analyzed the prevalence and correlation of traumatic stress symptoms associated with the COVID-19 pandemic among older people [32]. An analysis of the data collected by Horowitz and Wilner [33] was conducted on a sample of 3012 people aged 60 years and over. Study participants were asked to participate in an online survey in which post-traumatic stress disorder symptoms were measured using the Impact of Event Scale (IES). This scale is one of the best-known tools for measuring PTSD. In addition, it has also been adapted for use in assessing the impact of COVID-19—the so-called Impact of Event Scale with modifications for COVID-19 (IES-COVID19) [34]. It consists of 15 statements that characterize reactions to traumatic events, and 7 of them relate to measuring intrusions, i.e., intrusive experiences and images, while the remaining 8 relate to the study of avoidance, i.e., avoidance of memories and feelings recalling the traumatic event. All are scored on a 4-point scale, where 0 means “not at all” and 5 means “often”. The point score is directly proportional to the symptoms of the trauma. The higher it is, the greater its symptoms [33,34]. The following results were obtained in the study: 36.5% of the respondents reported experiencing clinically significant symptoms of traumatic stress related to trauma, such as COVID-19, of which 27.4% of the cases of those participating in the study may develop PTSD. Moreover, relatively more severe symptoms were reported by women and older adults [32]. The results of the conducted study show the great need for psychological support for a significant number of older people with post-traumatic stress disorder.

A study of mental health related to the impact of the COVID-19 pandemic was also conducted in Greece by V. A. Nikopoulou, V. Holeva, E. Parlapani et al. The study involved 538 respondents who were asked to complete a questionnaire in which post-traumatic stress symptoms were measured using several different psychometric scales [35]. One of these was the Fear of COVID-19 Scale (FCV-19S), developed in 2020 and recognized in many countries around the world as a good psychometric tool for assessing fear of COVID-19 during a pandemic. It is a seven-item scale containing items related to feelings about COVID-19 disease. Responses to the mentioned items can be given using a five-item Likert-type scale expressing the degree of agreement, where 1 means “strongly disagree” and 5 means “strongly agree”. The higher the score on the FCV-19S, the higher the level of fear of COVID-19 [36,37]. The results obtained by the Greek authors were as follows: 32.7% of women and 7.8% of men participating in the study were classified as having elevated fear with post-traumatic stress symptomatology. The apparent discrepancy between the results of both sexes does not mean that women showed significantly higher levels of fear than men. This is because the sample size in the study differed significantly by gender—women constituted 4/5 of the study sample and therefore the two groups cannot be compared [35]. Furthermore, although the majority of respondents experienced normal levels of fear, this does not mean that the mental health risks caused by COVID-19 anxiety do not exist. On the contrary, efforts to protect the mental health of the population should be intensified.

Another study dedicated to analyzing the symptoms and correlates of post-traumatic stress disorder during the COVID-19 pandemic, conducted on the Chinese population, showed the ongoing global mental health crisis in the population. The study was conducted using an online questionnaire. After analyzing all the answers given, 338 of them were included in the study. Post-traumatic stress disorder symptoms among the respondents were assessed using the PTSD checklist for the DSM-5 classification described in the APA Diagnostic and Statistical Manual—the so-called PCL-5 checklist [38]. The post-traumatic stress disorder checklist is a commonly used measure of PTSD symptoms according to the DSM-5 and consists of 20 items assessing the severity of PTSD according to the criteria in the DSM-5 classification, with respondents rating each of the listed problems on a five-point Likert scale from 0, which means “not at all”, to 4, which means “intensely”. The purpose of the list is to indicate the extent to which each symptom has bothered the survey participants over the past month. The total symptom severity score was obtained by adding up the scores for each of the 20 items—this ranges from 0 to 80, with the PCL-5 cut-off score according to preliminary studies being between 31 and 33, indicating that PTSD is likely to develop [39,40,41]. In the cited study, the analysis revealed that the mean PCL-5 score among respondents was 12.9. Additionally, 3.5% of the total sample reported a sum of PTSD symptoms above the PCL-5 cut-off point, which may speak to the development of PTSD. In contrast, 25.44% of the total sample met two or more criteria for a PTSD diagnosis, albeit with total PCL-5 scores below the cut-off point. Examining the PTSD symptom network was also an important part of the study. In the symptom network associated with the COVID-19 pandemic, self-destructive and reckless behaviors appeared to be the most central symptom [38]. 

The next scientific paper I cited examined the eating behavior of young adults in the German population for significant changes in the COVID-19 pandemic. The study involved 1980 Bavarian university students who were asked to complete a questionnaire. The authors obtained the following results: 610 people declared that they were eating more, while 328 people answered that they were eating less compared to before the pandemic started, and the remaining people did not change the amount of food they consumed. Furthermore, the presence of a correlation between increased alcohol consumption (42.3%), smoking (42.0%), psychological stress (35.4%), and eating more food during the COVID-19 pandemic was noted. These changes are undoubtedly linked to the introduction of restrictions in Bavaria, as well as the effect of increased anxiety and psychological stress affecting a great number of people during the pandemic. Indeed, in addition to the threat of potential infection, a pandemic also causes a high degree of uncertainty, which in turn increases feelings of agitation and hyper-vigilance, which can affect eating habits. This thesis is supported by the results of the authors of this study, who reported in one of their conclusions that a higher risk of consuming food in increased amounts is found in people who experience psychological stressors [42]. The conclusions of the cited study are very worrying because improper eating habits affect the health of people to a significant extent. Especially the problem of being overweight and obesity increases the risk of cardiovascular diseases, which have been the leading cause of death in the world for more than 20 years. Obesity, on the other hand, greatly increases the risk of developing severe COVID-19 pneumonia [43,44].

Another study worth mentioning concerns the mental-health status of COVID-19 survivors. The study was conducted in Pakistan and published on 6 January 2022 and is therefore highly relevant. Additionally, COVID-19 survivors are a particular group of people at risk of developing symptoms of PPSD, so it is particularly worth looking at this publication. The study group consisted of 70 people aged 18–60 years. To collect data the authors used tools to measure mental state, such as Impact Event Scale-Revised (IES-R), Patient Health Questionnaire-9 (PHQ-9), and Coronavirus Anxiety Scale (CAS) [45]. The former, the IES-R scale, is used to measure PTSD, consists of 22 items and takes into account its three different dimensions: intrusion in the form of excessive thoughts, avoidance, and overstimulation. Each of these statements is scored using a 5-point Likert-type scale, where 0 means “not at all” and 4 means “very much”. The cut-off score is 33 and indicates a high risk of PTSD symptomatology [46]. In turn, the PHQ-9 questionnaire is used for the initial diagnosis of depression and to measure the severity of its symptoms. As a measure of severity, the PHQ-9 score can range from 0 to 27, as each of the 9 criteria can be scored from 0 meaning “not at all” to 3 meaning “almost every day” [47,48]. In contrast, the CAS scale is a tool for measuring mental health and assessing dysfunctional anxiety associated with COVID-19. It takes the form of a 5-item test in which each item indicates a different physiological response to fear caused by the COVID-19 pandemic over the past 2 weeks. In response to each item listed, an appropriate rating is selected using a five-point scale, where 0 means “not at all” and 4 means almost every day” [49,50]. The authors of the above-mentioned study measured the level of depression among the respondents using the PHQ-9. The results obtained speak for a mass occurrence of depressive disorders among the recovered. Among all 70 people participating in the study, only 8 of them (11.4% of the total) did not show symptoms indicative of depression. Of the remaining 62 people, 27 of them (38.5% of the total) had mild depression and 18 (25.7% of the total) had moderate or severe depression. Using the IES-R scale, the authors then examined the prevalence of PTSD among participants who, as mentioned, had experienced COVID-19. The results show that as many as 47 respondents (67.1% of the total) manifested symptoms of post-traumatic stress disorder, while the remaining 23 respondents (32.9%) showed no effect of COVID-19 on their mental health. In turn, using the CAS scale, the authors measured the level of anxiety and fear associated with the COVID-19 pandemic. The results are as follows: 52 people (74.3% of the total) did not declare anxiety, while 14 people (29.7% of the total) showed anxiety related to COVID-19. Moreover, it was shown that patients with symptoms of COVID-19 have significantly higher levels of depression, stress, as well as anxiety, compared to asymptomatic patients [45]. This study shows the trauma faced by COVID-19 survivors and the need for effective psychological support. 

One study conducted this time in a Mexican sample also demonstrated psychological distress and the presence of post-traumatic stress symptoms in response to a health risk associated with COVID-19. The total study sample consisted of 3932 individuals. The previously mentioned IES-R scale was used to determine the presence of psychological distress in response to the traumatic event. The overall results obtained by the authors related to the frequency of psychological stress showed that 1160 people (27.7% of the total) had clinically significant symptoms of post-traumatic stress disorder. Regarding the presence of moderate or severe mental stress, 943 people (22% of the total) manifested intrusive thoughts, 933 people (22.3% of the total) demonstrated avoidance, while 515 people (12.2% of the total) exhibited excessive agitation. The results obtained are very worrying, as almost 30% of the respondents had symptoms of PTSD, which, for about a relatively large sample of the surveyed population, is a considerable percentage. In such a case, it seems justified to introduce all measures aimed at reducing the occurrence of PTSD symptoms. These may include activities, such as psychotherapy, including learning to manage emotions or psychoeducation [51].

## 5. The Mental Condition of Health Personnel and the COVID-19 Pandemic—Review of Conducted Research

The COVID-19 pandemic undoubtedly had a significant impact on the functioning of the health system. One study examined how the global health crisis associated with the pandemic affected the mental health of healthcare workers. The study of Spilg et al. [52] involved 962 Canadian medical professionals who were asked to complete an online survey. A series of dedicated scales and questionnaires were used to collect data, such as the MMD-HP, RMRS, and GAD-7. The first of these scales is the Revised Measure of Moral Distress for Healthcare Professionals (MMD-HP), which consists of 27 items. In it, participants rate each listed item according to a Likert scale in terms of how often it occurs in their practice, and how distressing it is when it occurs. The frequency score is then multiplied by the distress score, resulting in an overall score. The higher the score, the higher the level of moral distress [53,54]. The second is The Rushton Moral Resilience Scale (RMRS), which consists of 17 items. In it, participants rate the extent to which they agree with the listed items according to a Likert scale. A total score is obtained by calculating the average of all items. The higher the score, the greater the moral resilience [55]. The third item is the Generalized Anxiety Disorder (GAD) Scale, which is one of the basic tools to assess the level of anxiety and the likelihood of generalized anxiety disorder. It consists of 7 items and, similar to the previous ones, is based on a four-point Likert scale. Depending on the frequency of occurrence of a particular phenomenon in the past 14 days, participants can obtain from 0 to 3 points. Obtaining a minimum of 10 points is equivalent to a high probability of generalized anxiety disorder [56,57]. In the aforementioned study, one of the conclusions drawn by the authors was to determine the existence of a relationship between moral distress and professional exposure related to the care of patients infected with COVID-19. Based on the analysis of the results, it was defined that a higher percentage of medics having professional contact with patients infected with COVID-19 have been diagnosed with mental-health disorders compared to medics who did not have such contact. Furthermore, one of the conclusions was that relatively higher moral resilience was guaranteed by factors, such as older age, male gender, absence of mental disorders, and adequate psychological support from both colleagues and employer. Additionally, higher moral resilience translates into lower symptoms of anxiety, stress, and depression. In summary, any actions aimed at improving the psychological well-being of healthcare workers should primarily include interventions that address the root cause of anxiety and distress, which is their moral dimension, rather than simply counteracting these symptoms [52].

Another cited study examined resilience and subjectively perceived stress among paramedics both before and during the COVID-19 pandemic using tools, such as the Perceived Stress Questionnaire (KPS) and the Stress Measurement Scale (SPP-25) [58]. The KPS consists of 27 items assessing the level of the general sense of stress by considering its three dimensions in separate subscales. These include emotional tension, external stress, and intrapsychic stress. In addition, the questionnaire also includes a so-called lie scale. Respondents are asked to select an answer to each of the highlighted items according to a five-point Likert-type scale, where 1 means “not true” and 5 means “true”. The higher the total score obtained, the higher the stress level of the person tested [59]. The SPP-25, on the other hand, is a scale consisting of 25 items that describe the general level of resilience, which is a human personality trait. Some of the five factors included in it are tolerance of negative emotions, perseverance, determination, and the ability to mobilize in difficult situations. Each of these items is rated by the respondent according to a five-point Likert-type scale, where 0 means “definitely not” and 4 means “definitely yes”. The higher the score obtained, the similarly higher the level of resilience, and thus greater resilience [60]. The study involved Polish paramedics from the Pomeranian region working in the Hospital Emergency Department (ED) and the Polish Air Ambulance (LPR). The study group consisted of 84 persons. The authors of the study obtained the following results: paramedics having contact with COVID-19-infected patients showed a medium level of stress in comparison with paramedics without such contact, who were characterized by a low level of stress. The mentioned results refer to each of the KPS dimensions, i.e., external stress, intrapsychic stress, as well as emotional tension. Furthermore, it was shown that determination and perseverance were the most important factors in preventing an increase in perceived stress. Undoubtedly, experiencing stress daily in the work environment contributes to building psychological resilience. Therefore, having some kind of individual resilience resource by a professional group, such as paramedics, is extremely important to effectively cope with stress at work [58].

Another study determined the interaction of post-traumatic stress, depressive and anxiety symptoms on health professionals during the COVID-19 pandemic. A total of 514 medics working on the frontline of the pandemic in Italy participated in the study. Depressive, anxiety, and post-traumatic stress disorder symptoms were examined using diagnostic and psychometric tools, such as the IES-R, PHQ-9, and GAD-7, all of which have been described in previously cited publications. The results obtained by the authors were very unfavorable. As many as 115 respondents (23.5% of the total) manifested severe post-traumatic stress disorder. These people scored more than or equal to 10 points on the GAD-7 scale, which is equivalent to a high probability of generalized anxiety disorder. In addition, 99 respondents (20.2% of the total) had PHQ-9 scores equal to or greater than 10, indicating the existence of moderate to severe depressive disorders. Additionally, IES-R scale scores were higher for medics working in emergency departments compared to medics working in other departments. This means that medics working in emergency departments are relatively most affected by PTSD symptoms. This may be due, among other things, to the greater unpredictability of all the clinical cases occurring there, including patients with COVID-19. The authors of the study also determined in one of the results that anxiety, depressive symptoms, and PTSD were the main factors in the impairment of health professionals, which certainly harms their quality of life as well as patient care itself. These findings undoubtedly support the recognition of medical professionals as a particularly high-risk group for the development of mental-health disorders in the aftermath of the COVID-19 pandemic. There is no doubt that this professional group should have adequate access to psychological and psychiatric assistance. This is as important as fighting and countering the COVID-19 pandemic because only medics in a state of so-called mental well-being can adequately care for patients [61].

The mental and physical health of health professionals during the COVID-19 pandemic was also studied by B. Chang and A. Shechter. More specifically, the prevalence of psychological distress in medics working in emergency departments during the COVID-19 pandemic was assessed. The study group consisted of 52 medics who had professional contact with COVID-19-infected patients. In this study, dedicated tools were used to measure COVID-19 pandemic-related stress and PTSD using the PCL-5 scale, depression using the PHQ-9 scale, and anxiety using the GAD-7 questionnaire. All of these diagnostic scales have been described in more detail in previously discussed studies. The positive results obtained by the authors in the present study in terms of psychological symptoms appeared to be widespread among the respondents. Namely, 48% of all respondents experienced acute stress, 37% had depressive symptoms, and 30% had anxiety disorders. This is a kind of confirmation that medics experience severe stress in the work environment during the COVID-19 pandemic, thus simultaneously suggesting and highlighting the need for health professionals to receive appropriate psychological support [62].

The next study cited examined pandemic-dependent mental-health risks of COVID-19 among medical personnel working in the United States. A total of 571 participants were included in the study and asked to complete an online survey. The PTSD checklist for the DSM-5 classification, the PCL-5 checklist, was used to assess acute traumatic stress, the Patient Health Questionnaire-8 (PHQ-8) was used to assess depression, and the Generalised Anxiety Disorder Scale-7 (GAD-7) was used to assess anxiety. The risk threshold for the development of acute post-traumatic stress disorder according to the PCL-5 was at least 33, while the risk threshold for the development of depression according to the PHQ-8 was greater than or equal to 10, and the risk threshold for the development of anxiety according to the GAD-7 was 10. Taking into account the risk thresholds, the results obtained by the authors were as follows: 15% of all respondents were positive about the occurrence of acute traumatic stress, 20% of the total indicated the occurrence of depression, and 17% of the respondents indicated experiencing anxiety. In addition, it was estimated that medics working in emergency departments were more likely to develop these disorders compared to staff working in other hospital wards. This certainly contributes to the fact that medics working in emergency departments had a higher rate of contact with COVID-19-infected patients. The study indisputably showed that many medical professionals during a pandemic are at risk of developing disorders that impair their mental health. Therefore, professional groups working on the “front line” of the fight against a pandemic should be guaranteed access to various types of mental-health programs [63].

Post-traumatic stress disorder and health-worker depression during the COVID-19 pandemic were also screened in the population of England. The online screening was completed by a total of 103 health professionals. PTSD symptoms were measured by the authors using the PCL-5, while depression symptoms were measured using the PHQ-9 questionnaire [64,65]. After analyzing the results of the online survey, the authors contacted participants who scored above the cut-off for the PCL-5 or PHQ-9. The results of the survey were as follows: 44% of respondents met the criteria for PTSD, while the criteria for major depression were met by 39% of respondents. Furthermore, 24% of all respondents indicated the presence of trauma related to the COVID-19 pandemic. In addition, 29% of the study sample declared the presence of suicidal thoughts or thoughts related to self-harm. The results presented here show a range of traumas experienced by healthcare workers linked to PTSD and depression. Thus, this is another study that justifies the strong need to focus on psychological support for this particular professional group [66].

## 6. Strengths and Limitations

There are still few expert papers summarizing the major findings on post-pandemic stress syndrome—highlighting the importance of the COVID-19 pandemic for mental health. Much of the work that has appeared to date on the topic of PPSD tends to be very superficial opinions that lack a scientific basis or address the thread considered in this review in a rather cursory manner. The authors are aware that in the face of such a large number of studies, important reports may have been omitted—also due to the assumed eligibility criteria. Nonetheless, it should be noted that every effort was made to ensure that this review was conducted fairly, taking into account large, multi-center research designs and highlighting the main research streams in contemporary health psychology and psychotraumatology, particularly in terms of PPSD and PTSD.

## 7. Conclusions

According to many experts in psychology and psychiatry, the destabilization caused by the coronavirus pandemic may not be as noticeable now as it will be after the pandemic period is over [67]. Undoubtedly, the fact that the surrounding reality is now standardized and normalized by many contributes to this. According to many researchers, the scale and degree of trauma experienced by the community will not be perceived by many until after the pandemic has ended. Undoubtedly, the end of the pandemic, and thus the recovery of the world and the complete removal of the restrictions in place, will not make people stop being affected by renewed coronavirus events. This is mainly true for healthcare workers and those particularly affected by the pandemic who have contracted COVID-19 or lost loved ones to the disease [68,69]. For this reason, individuals experiencing post-pandemic stress syndrome should be included in preventive measures. It is also important to implement early psycheducation in order to effectively deal with isolation, misinformation, and other issues in the future that prove to be major stressors during the COVID-19 pandemic.

## Data Availability

Not applicable.
